# Early Imaging and Molecular Changes with Neoadjuvant Bevacizumab in Stage II/III Breast Cancer

**DOI:** 10.3390/cancers13143511

**Published:** 2021-07-14

**Authors:** José Manuel López-Vega, Isabel Álvarez, Antonio Antón, José Juan Illarramendi, Antonio Llombart, Valentina Boni, María José García-Velloso, Josep María Martí-Climent, Luis Pina, Jesús García-Foncillas

**Affiliations:** 1 Department of Medical Oncology, Marqués de Valdecilla University Hospital, 39008 Santander, Spain; onclvj@humv.es; 2 Department of Medical Oncology, University Hospital Donostia, 20080 Donostia-San Sebastián, Spain; ISABELMANUELA.ALVAREZLOPEZ@osakidetza.eus; 3 Department of Medical Oncology, University Hospital Miguel Servet, 50009 Zaragoza, Spain; aantont@gmail.com; 4 Medical Oncology Department, Complejo Hospitalario de Navarra, 31008 Pamplona, Spain; jj.illarramendi.manas@navarra.es; 5 Department of Medical Oncology, Hospital Arnau de Vilanova, 46015 Lleida, Spain; allombart1@yahoo.com; 6 START Madrid CIOCC, Hospital Universitario HM Sanchinarro, 28050 Madrid, Spain; valentina.boni@startmadrid.com; 7 Nuclear Medicine Department, Clínica Universidad de Navarra, 31008 Pamplona, Spain; mjgarciave@unav.es; 8 Department of Medical Physics and Radiation Safety, Clínica Universidad de Navarra, 31008 Pamplona, Spain; jmmartic@unav.es; 9 Department of Radiology, Clínica Universidad de Navarra, 31008 Pamplona, Spain; ljpina@unav.es; 10 Translational Oncology Division, OncoHealth Institute, University Hospital “Fundación Jiménez Díaz”, Autonomous University of Madrid, 28040 Madrid, Spain

**Keywords:** bevacizumab, breast cancer biomarkers, dynamic contrast-enhanced magnetic resonance imaging, positron emission tomography, vascular endothelial growth factor receptor-2

## Abstract

**Simple Summary:**

New blood vessel formation (angiogenesis) has a crucial role in tumour growth and spread. Bevacizumab is an anticancer therapy that targets angiogenesis by inhibiting the vascular endothelial growth factor (VEGF) and is approved for the treatment of metastatic breast cancer. However, there are no validated methods for predicting which patients will respond to bevacizumab, although some have investigated whether a response can be predicted by using scanning (imaging) techniques that study tumour blood vessels or by using the levels of VEGF markers before treatment. In this study, we used a combination of imaging techniques and VEGF marker levels to show that bevacizumab caused structural and functional changes in the blood vessels of breast tumours and substantially slowed tumour growth. The increasing availability and refinement of imaging technology can help to identify biomarkers that will be able to predict which patients with breast cancer are most likely to respond to bevacizumab.

**Abstract:**

This prospective, phase II study evaluated novel biomarkers as predictors of response to bevacizumab in patients with breast cancer (BC), using serial imaging methods and gene expression analysis. Patients with primary stage II/III BC received bevacizumab 15 mg/kg (cycle 1; C1), then four cycles of neoadjuvant docetaxel doxorubicin, and bevacizumab every 3 weeks (C2–C5). Tumour proliferation and hypoxic status were evaluated using ^18^F-fluoro-3′-deoxy-3′-L-fluorothymidine (FLT)- and ^18^F-fluoromisonidazole (FMISO)-positron emission tomography (PET) at baseline, and during C1 and C5. Pre- and post-bevacizumab vascular changes were evaluated using dynamic contrast-enhanced magnetic resonance imaging (DCE-MRI). Molecular biomarkers were assessed using microarray analysis. A total of 70 patients were assessed for treatment efficacy. Significant decreases from baseline in tumour proliferation (FLT-PET), vascularity, and perfusion (DCE-MRI) were observed during C1 (*p* ≤ 0.001), independent of tumour subtype. Bevacizumab treatment did not affect hypoxic tumour status (FMISO-PET). Significant changes in the expression of 28 genes were observed after C1. Changes in vascular endothelial growth factor receptor (VEGFR)-2p levels were observed in 65 patients, with a > 20% decrease in VEGFR-2p observed in 13/65. Serial imaging techniques and molecular gene profiling identified several potentially predictive biomarkers that may predict response to neoadjuvant bevacizumab therapy in BC patients.

## 1. Introduction

Vascular endothelial growth factor (VEGF) is a well-characterised and potent regulator of vascular development and angiogenesis whose effects arise mostly through binding to the VEGF receptor-2 (VEGFR-2) [1,2]. Angiogenesis has been shown to have a crucial role in tumour growth and metastases, and overexpression of VEGF is commonly observed in a variety of tumours, including breast cancer (BC) [3,4,5]. Tumour-expressed VEGF is particularly attractive as a target for anticancer therapy, and as such, a variety of agents aimed at blocking VEGF or its receptor have been developed for the treatment of cancer [6].

Bevacizumab is a recombinant, humanised, monoclonal antibody that binds to all isoforms of the VEGF subtype A (VEGFR-A). Bevacizumab was the first antiangiogenic drug to be approved for use in several solid tumours, including metastatic BC [7,8], and it has been shown to improve progression-free survival (PFS) and/or overall response rate (ORR) when used in combination with chemotherapy in several randomised controlled trials in patients with BC [9,10,11,12,13].

Despite the fact that several systemic biomarkers have been studied in BC, there are currently no validated methods for predicting response to bevacizumab treatment. Several noninvasive imaging techniques have been previously used as prognostic indicators in patients with solid tumours, including BC [14,15,16,17,18]. ^18^F-fluoro-3′-deoxy-3′-L-fluorothymidine (FLT)-positron emission tomography (PET) has been reported as predictive of therapy response in BC [14], and studies have correlated ^18^F-fluoromisonidazole (FMISO)-PET uptake with direct oxygen measurements, highlighting its value as a surrogate marker of hypoxia in various malignancies [15,16]. Dynamic contrast-enhanced magnetic resonance imaging (DCE-MRI) has also been effectively used in the study of tumour vasculature and is a potential imaging biomarker for assessing anticancer treatment [17].

With regard to molecular biomarkers, baseline plasma VEGF-A and VEGFR-2 levels have shown potential predictive value in BC in randomised phase III trials of bevacizumab [19,20,21,22]. A pilot study of 21 patients with untreated inflammatory BC showed that bevacizumab decreased activated VEGFR-2 levels in tumour cells, increased tumour apoptosis, and reduced tumour blood perfusion, as measured by DCE-MRI [23].

Based on these observations, it was postulated that tumour imaging profiles before and after bevacizumab treatment, combined with the status of circulating biomarkers, may be useful in the assessment of treatment efficacy and could provide insights into the mechanism of action of bevacizumab. The main goal of the present phase II study was to determine the clinical value of noninvasive tumour imaging techniques and molecular biomarker analysis in predicting the efficacy of neoadjuvant bevacizumab treatment in patients with locally advanced BC.

## 2. Results

### 2.1. Patient Characteristics

In total, 73 patients were enrolled in this study between 2009 and 2010; 72 met the inclusion criteria, of which two discontinued the treatment due to severe allergic reaction to docetaxel. Efficacy was therefore evaluated in 70 patients. The majority of tumours assessed in the enrolled patients at baseline were clinically staged as T2 (79.5%) with an axillary node involvement (58.9%) and infiltrating ductal carcinomas (86.3%; Table 1). Most patients had tumours with a high Ki-67 proliferative index (67.1%) but did not overexpress human epidermal growth factor receptor-2 (HER2; 76.7%; Table 1). Four BC subtypes were identified: triple-negative BC (TNBC; oestrogen receptor [ER]-, progesterone receptor [PgR]-, and HER2-negative; *n* = 11), HER2-positive (*n* = 14), luminal A (ER-positive, low-grade, and low proliferative index; *n* = 16), and luminal B (ER-positive, high-grade, and high proliferative index; *n* = 29; Table 1).

### 2.2. Treatment Efficacy and Response

Of the 70 patients analysed, 52 (74.3%) were considered to be responders (grade [G] 3/G4/G5). At the end of treatment, 7 patients (10.0% of the total) had a complete response (G5), 13 (18.6%) attained G4, 32 (45.7%) attained G3, and a G2/G1 response was achieved in 18 patients (25.7%), nine for each of the two grades.

Tumour grade at baseline was significantly associated with response (*p* = 0.006). Specifically, 10/18 (55.6%) nonresponders had a G1 tumour at baseline, 7/18 (38.9%) had a G2 tumour, and 1/18 (5.6%) had a G3 tumour, whereas 43/52 (82.7%) responders had a G2/G3 tumour and 9/52 (17.3%) had a G4 tumour. A nonsignificant association between response to bevacizumab treatment and clinical BC subtype was also observed (*p* = 0.06). All patients with TNBC at baseline responded to treatment (*n* = 11), while nine and one patients with luminal A and B tumours, respectively, were considered responders. Of the 14 patients with ER-negative tumours, eight (57%) were in the best-response group (*p* = 0.01).

#### 2.2.1. Clinical Value of Noninvasive Tumour Imaging Techniques

FLT-PET imaging detected tumours in 67 patients (95.8%) at baseline, and FLT uptake was significantly higher in stage III (*p* = 0.006), G3 (*p* = 0.001), and TNBC (*p* = 0.004) versus other BC subtypes (Table 2). FLT-PET maximum standardised uptake values (SUV_max_) significantly correlated with Ki-67 expression (ρ = 0.38, *p* = 0.001). In all samples studied, there was a significant reduction in FLT tumour uptake (FLT SUV_max_ and tumour to tissue ratio [TTR]) and proliferative tumour activity (PTAc; *p* < 0.001) from baseline to cycle (C) 1 (Table 3), which was independent of BC subtype. A > 25% decrease in FLT tumour uptake was seen in 36 patients (52.9%); only one patient (1.5%) was found to have a > 25% increase in tumour proliferation after bevacizumab treatment. It was also observed that tumours with a ≤ 25% decrease in FLT uptake showed higher baseline VEGFR-2 expression (*p* = 0.02).

At baseline, FMISO-PET-visible tumours were detected in 41/69 patients (56.2%; data unavailable for one patient), and FMISO uptake was significantly higher in stage III, G3, and TNBC tumours (Table 3). FMISO-PET SUV_max_ was significantly correlated with VEGFR-2 expression (ρ = 0.26, *p* = 0.02) but not with micro vessel density. Significant correlations were observed between FMISO at baseline (FMISO1), SUV_max_, and Ki-67 (ρ = 0.35, *p* = 0.006), and between FMISO1 and FLT1 SUV_max_ (ρ = 0.55, *p* < 0.001). FMISO uptake did not differ significantly before or after bevacizumab therapy or by BC subtype. However, median FMISO values were significantly different in G1 and G3 tumours (*p* = 0.03). Following bevacizumab treatment, hypoxia increased in G1 tumours (12.58%, interquartile range [IQR] 37.32) but decreased in G3 tumours (11.95%, IQR 17.39). Tumours with a > 25% decrease in hypoxia showed a significant reduction in the number of proliferative endothelial cells (*p* = 0.019).

**Figure 1:**
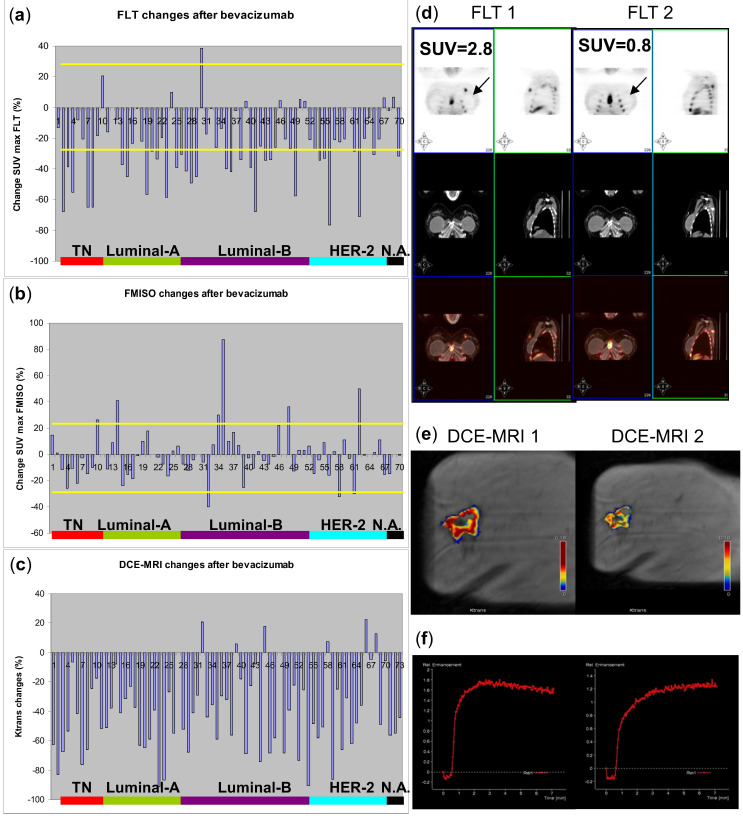
Waterfall plots of changes in (**a**) FLT and (**b**) FMISO SUV_max_, and (**c**) DCE-MRI K^trans^ at baseline and after treatment by clinical subtype in patients. Example of a patient showing similar levels of response in both (**d**) FLT-PET and (**e**,**f**) DCE-MRI parameters. (**d**) shows important decreases in FLT SUV_max_ after bevacizumab; (**e**) shows changes in the same patient in K^trans^ before (DCE-MRI 1) and after bevacizumab (DCE-MRI 2). DCE-MRI, dynamic contrast-enhanced magnetic resonance imaging; FLT, ^18^F- fluoro-3′-deoxy-3′-L-fluorothymidine; FMISO, ^18^F-fluoromisonidazol; HER2, human epidermal growth factor receptor 2; K^trans^, volume transfer constant; PET, positron emission tomography; SUV_max_, maximum standardised uptake; TNBC, triple negative breast cancer.

Significant decreases from baseline (*p* < 0.001) were observed in all DCE-MRI parameters at C1 (Table 3), irrespective of BC subtype or clinical-pathological features. Only area under the curve (AUC) had a mild but nonsignificant association with lymph node disease (*p* = 0.06). A correlation was found between changes in DCE-MRI parameters and FLT-PET changes at C1 (Figure 1), the most significant being between the volume transfer constant (K^trans^) and the flux rate constant (K_ep_), respectively, with FLT SUV_max_ (ρ = 0.414, *p* < 0.001 and ρ = 0.449, *p* < 0.001).

#### 2.2.2. Analysis of Gene Expression

Microarray analysis of 119 pre- and post-therapy samples (baseline and after C1 and C5) showed significant changes in gene expression after treatment. Downregulated genes (9/28) included delta-like 4 (*DLL4)*, *HEYL*, *FLT1*, angiopoietin-2 (*ANGPT2*), and endothelial cell-specific molecule 1 (*ESM1),* and overexpressed genes (19/28) included *HMOX1*, *CD163*, *PLTP*, and *DPP4*. We identified 61 Gene Ontology categories mainly involved in angiogenesis, immune response, and cell death (Table S1 and Figure S1). No differences in gene expression were observed after C5. We were unable to confirm these findings by polymerase chain reaction (PCR) analysis as there were no tumour samples available.

**Figure 2:**
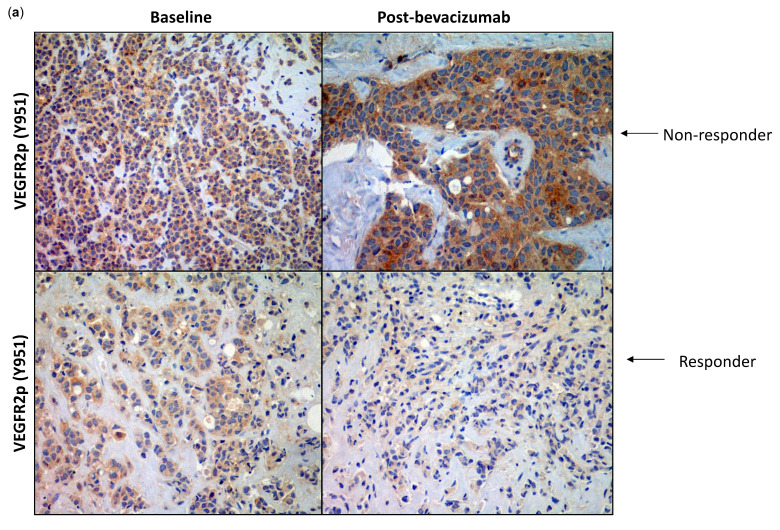

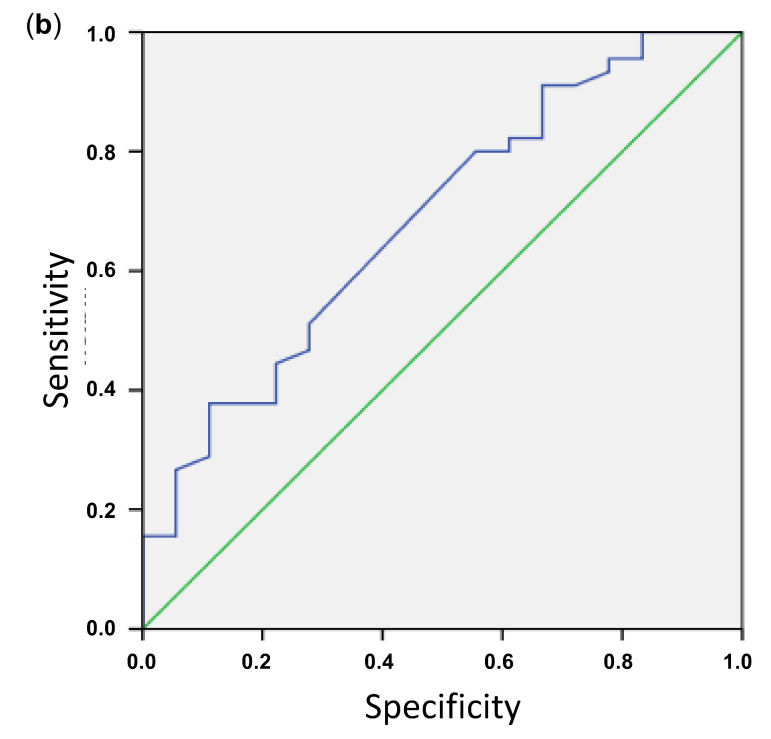
(**a**) Examples of representative immunohistochemistry staining of vascular endothelial growth factor receptor-2 (VEGFR-2) at baseline and post-bevacizumab for responders and nonresponders; (**b**) VEGFR-2 changes after bevacizumab treatment by immunohistochemistry staining and association with response as shown by receiver operator characteristic curve (AUC = 0.68, 95% CI 0.54, 0.82; *p* = 0.02). AUC, area under the curve; CI, confidence interval.

Changes in VEGFR-2p expression, the main receptor target of VEGF, were observed by immunohistochemistry in 65 patients (Figure 2a); 17 of these patients showed gross tumour residual disease, 18 had minimal residual disease, and 30 had intermediate disease. A decrease in VEGFR-2p expression of >20% was obtained in 13 patients (20%) after bevacizumab treatment (Figure 2b).

Significant differences in gene expression were found between BC subtypes. TNBC tumours showed overexpression of *VEGF-A*, *NOTCH1*, *CXCR4*, *IL8*, *SFRP1*, and *HIF1α*. Significant correlations were observed between FMISO uptake and *VEGF-A* (ρ = 0.475, *p* < 0.001), *HIF1α* (ρ = 0.347, *p* = 0.007), and *IL-8* (ρ = 0.363, *p* = 0.004) expression. An inverse correlation was also observed between *RHOB* expression and FMISO uptake (ρ = −0.46, *p* < 0.001), indicative of *RHOB* downregulation in more hypoxic tumours. Analysis of responders and nonresponders to FLT-PET revealed 12 genes that were significantly differentially expressed, including *PDGF-D*, which was found to be downregulated in tumours with a significantly decreased proliferation rate; these findings were confirmed by PCR analysis.

### 2.3. Treatment Safety

All 73 patients were included in the safety analysis. Patients received a median of five cycles of bevacizumab (mean 4.9, standard deviation [SD] 0.4) and four cycles of docetaxel and doxorubicin (mean 3.9, SD 0.4). No deaths were reported. Overall, 53 grade 3/4 adverse events (AEs) were reported (Table 4), with the most common being febrile neutropenia (29 events; 39.7% of grade 3/4 AEs). Incidence of AEs of special interest with bevacizumab, such as haemorrhage and hypertension, occurred with low incidence and were primarily grade 1 or 2. There were no reports of cardiac failure or thrombosis. Seven (10.8%) patients had complications in wound healing after surgery.

Treatment delays occurred in 23 (31.5%) patients, mostly due to neutropenia (43 episodes) or mucositis/stomatitis (four episodes). Dose reductions were needed in 2 (2.7%) patients while receiving bevacizumab, 13 (17.8%) patients while receiving docetaxel, and 11 (15.1%) patients while receiving doxorubicin. Granulocytic growth factor support was required in 41 (56.2%) patients.

## 3. Discussion

In this multicentre, prospective phase II study, neoadjuvant bevacizumab treatment induced structural and functional changes in BC-infiltrating vessels and substantially slowed tumour growth by decreasing tumour proliferation, as shown by our combined approach of noninvasive imaging and molecular biomarker analysis. The treatment also induced a significant decrease in VEGFR-2p levels and was associated with downregulation of the gene expression of several potentially predictive molecular biomarkers in patients with BC. Treatment was well tolerated, with no deaths reported during the study.

Imaging with FLT- and FMISO-PET and DCE-MRI has previously been used to evaluate potentially prognostic tumour characteristics in several malignancies, including BC [14,15,16,17]. Likewise, other imaging techniques are also known to have prognostic potential in specific malignancies, including head and neck cancer, non-small-cell lung cancer, colorectal cancer, and melanoma [18]. This study provides further evidence that these imaging techniques can effectively monitor tumour proliferation, hypoxia, and changes in tumour vasculature in patients with BC. These noninvasive approaches also show potential in determining the therapeutic efficacy of bevacizumab in the treatment of BC, for which there is no predictive biomarker for tumour response. Our research shows the potential of using imaging techniques to identify patients that could respond to neoadjuvant treatment with bevacizumab. Our combination of molecular biomarkers and imaging techniques may be difficult to set up for routine clinical practice. However, the use of, at least, MRI could greatly aid the identification of patients with potentially better response to bevacizumab in the neoadjuvant setting.

Data have shown that breast cancer patients with high baseline levels of serum VEGF (≥367 ng/mL) are more responsive to any type of breast cancer treatment than patients with lower levels and that serum VEGF is an independent predictor of treatment response [24]. Although some studies suggested that baseline plasma VEGF-A levels are predictive of bevacizumab response [19,20,21], the prospective MERiDian trial was unable to validate baseline plasma VEGF levels as a predictor of bevacizumab efficacy [25]. These data suggest that reliable predictors of bevacizumab response are likely to require a combination of biomarkers [26].

Bevacizumab affected tumour proliferation in the majority of BCs evaluated, and these effects were found to be independent of subtype. These results are consistent with a previous study [23], in which changes in tumour vascular permeability were observed in patients with primary BC receiving bevacizumab. Tumour hypoxia is a prognostic factor influencing response and survival in many malignancies [27,28,29] and an established indicator of poor clinical outcome in BC [30,31]. Like most tumours, BCs show regions of hypoxia, and adaptation to these hypoxic conditions eventually leads to increased tumour metastases. Importantly, hypoxic tumours have been shown to be resistant to chemotherapy and radiation therapy [32]. Our findings indicate that hypoxic tumour status in BC cells was not affected by bevacizumab treatment. This could possibly be due to the significant reductions in tumour proliferation and perfusion with bevacizumab, which may mask its effect on tumour vascularisation.

With regard to expression of molecular biomarkers, bevacizumab treatment led to a >20% decrease in VEGFR-2p levels in 20% of patients in this study. Bevacizumab was also associated with a downregulation in the expression of genes that encode several key mediators of the NOTCH signalling pathway, including *DLL4*, *HEYL*, *FLT1*, *ANGPT2,* and *ESM1*. Previous studies have shown that the migration of DLL4-expressing tip endothelial cells, a specialised subtype of endothelial cells that mediate the growth of vessels during angiogenesis, leads to new vessel sprouting and is accompanied by the proliferation of NOTCH1-expressing sprout stalk endothelial cells [33]. In the current study, bevacizumab treatment led to reduced *DLL4* expression and inhibited the NOTCH signalling pathway, as confirmed by the downregulation of the NOTCH target gene, *HEYL*. These findings support the hypothesis that bevacizumab exerts its antitumour effects through inhibition of VEGF, as well as the NOTCH signalling pathway, which may explain the disruption of tumour angiogenesis [33].

Downregulation of *ESM1* expression was also observed in the present study. Several studies have reported a correlation between ESM1 expression and angiogenic processes during tumour progression, with ESM1 being crucial for vascular growth through the extracellular matrix [34,35,36], and have shown that high ESM1 levels are associated with increased risk of BC metastasis [37]. Moreover, ESM1 enrichment has been described in tip cells [38], and *ESM1* downregulation characterizes the transcriptional switch of fast-growing angiogenic tumours to dormant tumours [39,40]. Therefore, decreased *ESM1* expression may be an important potentially prognostic biomarker in patients with BC receiving bevacizumab.

In this study, the ANGPT-TIE system was affected by bevacizumab, as confirmed by the downregulation of *ANGPT2* expression. ANGPT2 is secreted by endothelial cells at sites of active vasculature remodelling, promotes dissociation of pericytes from pre-existing vessels, and increases vascular permeability [41]. This study demonstrated a substantial reduction in vascular permeability with bevacizumab treatment, with decreases in K_ep_ and extravascular volume fraction (V_e_) on DCE-MRI. The changes in these parameters may be due to the inhibition of vascular remodelling induced by *ANGPT2* downregulation. Taken together with previous findings, the present study suggests that blocking VEGF signalling with bevacizumab inhibits vessel sprouting by reducing the expression of key mediators of the NOTCH signalling cascade, which may induce an angiogenic dormant tumour phenotype.

This study has several potential limitations. The study was designed to only assess the effects of bevacizumab on BC tumours, and further analyses to evaluate tumour response to neoadjuvant chemotherapy are warranted. In addition, identification of the best subgroup of patients who could benefit from bevacizumab was restricted by the small patient population and limited number of cases within each molecular subtype and histologic grade. It should also be noted that the imaging techniques used in this study may not be available in all cancer centres.

## 4. Materials and Methods

### 4.1. Patients

In this prospective, single-arm, multicentre, phase II trial conducted at 11 centres in Spain, chemotherapy-naïve patients with stage II/III BC received neoadjuvant bevacizumab combined with docetaxel and doxorubicin (ClinicalTrials.gov (accessed on 9 July 2021) identifier: NCT01338753). Eligible women were aged 18–70 years with an Eastern Cooperative Oncology Group performance status (ECOG PS) 0–1, left ventricular ejection fraction ≥50% without signs of heart failure, and adequate bone marrow and organ function. Clinical stage was defined according to the American Joint Committee on Cancer staging guidelines [42]. Patients with New York Heart Association classification ≥II heart disease in the last 6 months, metastatic disease, major surgery ≤28 days of the start of therapy, and/or a history of thrombosis were excluded.

The trial was approved by the institutional review board and the ethics committees at each participating site, and all patients provided written informed consent prior to any study-related procedure.

### 4.2. Study Design

Patients received bevacizumab 15 mg/kg intravenously on day 1 of C1, followed by bevacizumab plus docetaxel 60 mg/m^2^ and doxorubicin 50 mg/m^2^ on the first day of each subsequent cycle (C2–C5); cycles were repeated every 3 weeks until there was evidence of unacceptable toxicity or a request from the patient to withdraw. The initial bevacizumab dose (C1) was administered over 90 min; if well tolerated, the second infusion was delivered over 60 min (C2) and subsequent infusions over 30 min (C3–C5). Postoperative radiation and systemic therapy were administered according to standard criteria [43].

### 4.3. Clinical Assessments

Patients underwent complete medical examinations, including ECOG PS, blood pressure, complete blood counts, routine serum chemistry, urinalysis, cardiac function, and chest X-rays or computed tomography (CT) scans. Tumour biopsies were collected at baseline (≤14 days before the start of C1) and during C1 and C5 (12–19 days after day 1 of respective cycles, if feasible; and before surgery in C5). Radiographic tumour assessments were performed at baseline. Tumour proliferation and hypoxia were assessed using FLT- and FMISO-PET, respectively, at baseline (≤14 days before the start of C1) and during C1 and C5 (12–19 days after day 1), as used in a previous study of bevacizumab in patients with locally advanced BC [23]. Vascular tumour changes were investigated with DCE-MRI. AEs were reported according to MedDRA v13.0 and classified using the National Cancer Institute Common Terminology Criteria v3.0 [44].

### 4.4. Treatment Efficacy

Pathological response in the primary tumour was evaluated post-surgery and was scored as G1 (no change/some alteration to individual tumour cells but no reduction in overall cellularity); G2 (≤30% loss of tumour cells but overall cellularity still high); G3 (30–90% reduction in tumour cells); G4 (>90% loss of tumour cells; only small clusters or widely dispersed individual cells remain); or G5 (no tumour cells detected by microscopic evaluation from the site of the tumour; vascular fibroelastotic stroma often with infiltrating macrophages and ductal carcinoma in situ might be present) [45].

### 4.5. Positron Emission Tomography

PET/CT scans were performed centrally at baseline and during C1 and C5. Patients remained in the study irrespective of FLT tumour uptake during the baseline scan. Images were acquired on a hybrid scanner (Biograph Duo; Siemens/CTI; Malvern, PA, USA) and were evaluated by two nuclear medicine physicians blinded to study results. In cases where PET scan could not determine tumour volume, a CT scan was used. For quantitative tumour uptake, SUV_max_ was calculated with eSOFT software (Siemens Medical Solutions; Siemens/CTI, Malvern, PA, USA) using the single maximum pixel count within the volumes of interest (VOIs) placed in the area showing highest tumour activity [46]. TTR was calculated for both FLT- and FMISO-PET. PTAc and hypoxic tumour activity (HTAc) were calculated by multiplying volume of the lesion with its corresponding mean SUV of FLT and FMISO. Volume of each lesion was calculated using an automated contouring program based on the SUV using a threshold of 50% of SUV_max_. Details of PET scan reconstruction and FLT synthesis and quantification are provided in Appendix A.

### 4.6. Dynamic Contrast-Enhanced Magnetic Resonance Imaging

DCE-MRI was performed centrally at baseline (≤14 days before the start of C1) and during C1 and C5 (12–19 days after day 1) on a 1.5 T GE system (GE Healthcare; Waukesha, WI, USA). Three sets of baseline images were obtained before intravenous contrast administration (Magnevist, gadolinium dimeglumine pentatate; Berlex Laboratories; Whippany, NJ, USA). Images were analysed using an IDL-based analysis program (IDL Corp; Dallas, TX, USA) and a modified version of Cine Tools (GE Healthcare, Waukesha, WI, USA). Using a two-compartment model [47], the following parameters were obtained: K^trans^, K_ep_, V_e_, and AUC, defined by the early and late enhancement assessed at 90 and 180 s, respectively.

### 4.7. Immunohistochemistry

Ultrasound-guided tumour biopsies were obtained using a vacuum-assisted 12-gauge device (Celero, Hologic; Marlborough, MA, USA) at baseline and during C1 and C5 (12–19 days after day 1). The samples were fixed in formalin and embedded in paraffin and analysed in a single laboratory. Immunohistochemistry was performed on 3 µm sections using the EnVision^TM^ + System Peroxidase/DAB (Dako; Glostrup, Denmark). Proliferative endothelial cells were visualised using the specific endothelial marker CD31 and Ki-67. CD31 was visualised using the MACH 2 Mouse AP-polymer (MALP521G, Biocare Medical Inc.; Pacheco, CA, USA) and Vulcan Fast Red (FR805H, Biocare Medical Inc., Pacheco, CA, USA). CD31-positive cells were visualised using red cytoplasmic staining, and Ki-67-positive cells using brown nuclear staining. Microscopic assessment of CD31 staining was used to evaluate micro vessel density, and the Chalkley count technique was used to quantify angiogenesis in the tumour sections stained with anti-CD31 [45]. Staining scores for other immunohistochemistry biomarkers, including ER, PgP, and HER2, were established independently by two observers who were blinded to clinical data using the H-score method.

### 4.8. Gene Profiling

Tumour biopsies were collected in RNA later^®^ solution (Ambion; Foster City, CA, USA). Total RNA was isolated using an Ultra Turrax T25 homogeniser and extracted using the Trizol^®^ protocol following the RNeasy^®^ kit (Qiagen; Germantown, MD, USA). The quantity and quality of RNA were assessed using a NanoDrop spectrophotometer (NanoDrop Technologies; Wlimington, DE, USA). RNA quality was assessed only for samples with ≥450 ng RNA using the Agilent 2100 Bioanalyzer (Agilent Technologies; Mulgrave, VIC, Australia). Gene profiling of samples was performed at baseline and during C1 and C5 (12–19 days after day 1; see Appendix A for further details regarding gene profiling analysis). Genes were selected as significant using a B statistic cut off (B > 0). Due to the patient heterogeneity, an additional paired *t*-test was performed with 55 paired patients and a false discovery rate (FDR) of 0.01 was established as selection criteria [48]. The normalization procedure and statistical data analyses were performed with Bioconductor [49].

### 4.9. Statistical Analysis

The study was designed to enrol 73 patients in order to detect a significant change from baseline to the end of C1 in each parameter that was equal to one SD of the change, with a power of 95% at a significance level of α = 0.05 using two-sided Wilcoxon signed-rank test (or paired *t*-test).

The Mann–Whitney U test was used for between-group comparisons. For paired samples, the Wilcoxon signed-rank test was applied. Spearman correlations were computed to assess the association between two variables. Most continuous variables followed a nonparametric distribution (Kolmogorov–Smirnov and Shapiro–Wilk test, *p* < 0.05). Association between PET/CT, DCE-MRI, and clinical-pathological features was estimated using Mann–Whitney and Kruskal–Wallis tests. Association between categorical variables was estimated using the χ^2^ test. Correlations between imaging parameters and biomarkers were calculated using the Spearman’s ρ test. All statistical tests were performed using SPSS software v15.0 for Windows (SPSS, Inc., Chicago, IL, USA) and regarded as statistically significant if *p* < 0.05.

## 5. Conclusions

A combination of serial imaging techniques and molecular gene profiling indicates that several potentially predictive biomarkers may be used to monitor the efficacy of neoadjuvant bevacizumab therapy in patients with BC. As the availability of noninvasive imaging techniques increases, the identification of these predictive biomarkers for bevacizumab efficacy may play an important role in BC management.

## Figures and Tables

**Table 1:** Baseline patient and tumour characteristics.

Characteristics		Patients
		(*n* = 73)
Mean age, years (SD)		48.3 (9.8)
Menopausal status		
Pre		32 (43.8)
Post		9 (12.3)
Amenorrhoea > 2 years		11 (15.1)
N/A		21 (28.8)
Histopathological type		
Ductal		63 (86.3)
Lobular		3 (4.1)
Mixed ductal and lobular		3 (4.1)
Mucinous		1 (1.4)
Adenocarcinoma NOS		2 (2.7)
Primary occult tumour		1 (1.4)
Histopathological grade		
G1		19 (26.0)
G2		37 (50.7)
G3		16 (21.9)
Gx		1 (1.4)
Tumour size		
T2		58 (79.5)
T3		14 (19.2)
Tx		1 (1.4)
Lymph node status		
N0		30 (41.1)
N1		36 (49.3)
N2		7 (9.6)
Clinical stage IIA IIB IIIA		
	(T2N0)	23 (31.5)
	(T2N1)	30 (41.1)
	(T3N0)	7 (9.5)
	(TxN2)	1 (1.3)
	(T2N2)	5 (6.8)
	(T3N1)	6 (9.5)
	(T3N2)	1 (1.3)
HER2 status		
HER2+		14 (19.2)
HER2−		56 (76.7)
N/A		3 (4.1)
Ki-67 proliferation index		
High (>14%)		49 (67.1)
Low (<14%)		22 (30.1)
Clinical subtypes		
Triple negative		11 (15.1)
HER2 positive		14 (19.2)
Luminal A		16 (21.9)
Luminal B		29 (39.7)

Gx, grade unknown; HER2, human epidermal growth factor receptor-2; Ki-67, proliferation index; N/A, not available; NOS, not otherwise specified; SD, standard deviation; Tx, size unknown. Data are presented as number (%) unless otherwise specified.

**Table 2:** Association between baseline clinical-pathological characteristics and FLT uptake.

Tumour Characteristics		FLT SUV_max_	
		Median (IQR)	*p* Value
Size	T2	2.75 (1.58–3.92)	0.577
	T3	2.94 (2.11–4.86)	
Lymph node status	N0	2.13 (1.38–3.60)	0.036
	N1	2.81 (1.87–3.93)	
	N2	3.95 (3.47–5.99)	
Clinical stage	II	2.62 (1.53–3.77)	0.006
	III	3.81 (2.87–5.34)	
Tumour grade	G1	2.11 (1.30–2.90)	0.001
	G2	2.59 (1.73–3.92)	
	G3	3.99 (3.40–5.42)	
ER status	Positive	2.69 (1.55–3.78)	0.009
	Negative	3.72 (3.06–5.51)	
PgR status	Positive	2.72 (1.48–3.77)	0.07
	Negative	3.40 (2.15–4.21)	
HER2 status	Positive	2.78 (2.13–3.83)	0.649
	Negative	2.78 (1.47–4.09)	
Ki-67	<14%	2.03 (1.39–3.26)	0.029
	>14%	3.23 (2.12–4.30)	
Clinical subtype	Triple negative	4.15 (3.35–5.57)	0.004
	HER2 positive	2.78 (2.13–3.83)	
	Luminal A	1.67 (1.28–2.69)	
	Luminal B	2.94 (1.90–3.93)	

ER, oestrogen receptor; FLT, 18F- fluoro-3′-deoxy-3′-L-fluorothymidine; HER2, human epidermal growth factor receptor-2; IQR, interquartile range; Ki-67, proliferation index; PgR, progesterone receptor; SUVmax, maximum standardised uptake value.

**Table 3:** Changes in imaging parameters from baseline until the end of treatment after cycle 5.

Imaging Parameters	Pre-Bev, Median (IQR)	Post-Bev, Median (IQR)	Pre-Bev to Post-Bev, % Change Median (IQR)	*p* ^1^	Patients, *n*
PET					
FLT SUV_max_	2.78 (2.34)	1.85 (1.41)	−26.06 (25.85)	<0.001	68
FLT TTR	6.1 (6.5)	4.4 (3.59)	−17.42 (34.93)	<0.001	68
PTAc	16.85 (26.27)	10.56 (16.77)	−30.11 (35.79)	<0.001	63
FMISO SUV_max_	1.21 (0.49)	1.15 (0.48)	−1.89 (19.85)	0.289	66
FMISO TTR	1.04 (0.43)	1.08 (0.41)	−2.07 (22.77)	0.648	66
HTAc	13.53 (18.11)	10.36 (18.4)	−6.6 (59.31)	0.124	66
DCE-MRI					
K^trans^	115.25 (89)	58.5 (48.5)	−46.08 (37.96)	<0.001	70
K_ep_	260.75 (155.88)	158.5 (95)	−37.87 (34.51)	<0.001	70
V_e_	472.5 (187.5)	423 (212.5)	−10.95 (33.71)	0.001	70
AUC_60_	11.83 (9.32)	6.22 (6.25)	−46.14 (39.73)	<0.001	70

AUC_60_, area under the curve from 0 to 60 s; Bev, bevacizumab; DCE-MRI, dynamic contrast-enhanced magnetic resonance imaging; FLT, ^18^F-fluoro-3′-deoxy-3′-L-fluorothymidine; FMISO, ^18^F-fluoromisonidazole; HTAc, hypoxic tumour activity; IQR, interquartile range; K_ep_, flux rate constant; K^trans^, volume transfer constant; PET, positron emission tomography; PTAc, proliferative tumour activity; SUV_max_, maximum standardised uptake value; TTR, tumour to tissue ratio; V_e_, extravascular volume fraction. ^1^ Calculated using Wilcoxon paired test.

**Table 4:** Summary of grade ≥ 3 adverse events following treatment.

System Organ Class	Preferred Term	Grade 3, *n* (%)	Grade 4, *n* (%)	Total, *n* (%)
Blood and lymphatic system disorders	Febrile neutropenia	8 (11.0)	21 (28.8)	29 (39.7)
	Leukopenia	8 (11.0)	6 (8.2)	14 (19.2)
	Neutropenia	4 (5.5)	10 (13.7)	14 (19.2)
	Febrile bone marrow aplasia	1 (1.4)	-	1 (1.4)
GI disorders	Stomatitis	3 (4.1)	-	3 (4.1)
	Vomiting	3 (4.1)	-	3 (4.1)
	Diarrhoea	1 (1.4)	-	1 (1.4)
	GI mucositis	1 (1.4)	-	1 (1.4)
Infections/Infestations	H1N1 influenza	-	1 (1.4)	1 (1.4)
	Infection	1 (1.4)	-	1 (1.4)
	Vulvar abscess	1 (1.4)	-	1 (1.4)
General disorders	Asthenia	1 (1.4)	-	1 (1.4)
	Mucosal inflammation	1 (1.4)	-	1 (1.4)
Reproductive system/ breast disorders	Menstruation irregular	2 (2.7)	-	2 (2.7)
Immune system disorders	Drug hypersensitivity	1 (1.4)	-	1 (1.4)
Investigations	Blood potassium decreased	1 (1.4)	-	1 (1.4)
Skin/subcutaneous tissue disorders	PPES	1 (1.4)	-	1 (1.4)
Vascular disorders	Hypertension	1 (1.4)	-	1 (1.4)

AE, adverse event; GI, gastrointestinal; PPES, palmar-plantar erythrodysesthesia syndrome.

## Data Availability

The data presented in this study are available on request from Roche (see data sharing section). The data are not publicly available due to company restrictions.

## Supplementary Materials

The following are available online at https://www.mdpi.com/article/10.3390/cancers13143511/s1, Figure S1: Hierarchical clustering of 119 samples according to expression of the top 28 differentially expressed probes (pre- and post-bevacizumab), Table S1: Differential change in gene expression from baseline after bevacizumab treatment using Linear Models for Microarray data.

## Data Sharing

Qualified researchers may request access to individual patient level data through the clinical study data request platform (https://vivli.org/ (accessed on 9 July 2021)). Further details on Roche’s criteria for eligible studies are available here (https://vivli.org/members/ourmembers/ (accessed on 9 July 2021)). For further details on Roche’s Global Policy on the Sharing of Clinical Information and how to request access to related clinical study documents, see here (https://www.roche.com/research_and_development/who_we_are_how_we_work/clinical_trials/our_commitment_to_data_sharing.htm (accessed on 9 July 2021)).

## Appendix A

### Appendix A.1. PET Scanning and Reconstruction

Dynamic PET scans were carried out in an ECAT EXACT HR+ scanner (CTI/Siemens, Malvern, PA, USA). Patient was placed with the tumour centred in the field of view (15.2 cm). A 5-min transmission study with three ^68^Ge sources was conducted to perform the attenuation correction. Simultaneously with a bolus injection of 385 ± 56 MBq (10.4 ± 1.5 mCi) FLT, a dynamic acquisition was initiated in 2D mode with a total duration of 60 min, with a sequence 6 × 5 s, 6 × 10 s, 3 × 20 s, 5 × 30 s, 5 × 60 s, 8 × 150 s, 6 × 300 s. The reconstruction was performed as 128 × 128 matrices using ordered subset expectation maximisation (OSEM) with two iterations and eight subsets followed by a post-smoothing of the reconstructed image using a 5 mm FWHM Gaussian filter. Corrections were applied to account for scattered photons, random events, and photon attenuation. Each frame of the dynamic study had 63 axial slices [50].

Mean SUV were calculated using the average counts within threshold-defined volumes of interest that only included pixels greater than 40% (SUV_40_), 50% (SUV_50_), 60% (SUV_60_), 70% (SUV_70_), and 80% (SUV_80_) of the maximum value within a lesion. VOIs were placed in reference segments of the following organs: nontumour ipsilateral breast tissue, contralateral breast tissue, liver, mediastinum, subscapularis muscle, and spine bone marrow. SUV_max_ and mean SUV_50_ were calculated for each reference tissue.

FLT TTR ratios were obtained by FLT SUV_max_ ratio with the median value uptake of homolateral breast tissue that showed high correlation in pretherapy and post-therapy studies. FMISO TTR ratios were obtained by FMISO SUV_max_ ratio with the mediastinum or muscle median value uptake. We considered that TTR ratio was better to compare in paired studies before and after bevacizumab treatment for reducing intravariability due to the method. PTAc and HTAc parameters allowed us to consider intratumoural heterogeneity and tumour size.

### Appendix A.2. FLT Synthesis

FLT was synthesised and prepared for human use at the University Clinic of Navarra. Syntheses and quality control strictly followed the standard operating procedures and were subject to inspection. The labelling yield, radiochemical purity, and specific radioactivity of FLT were checked and recorded after each production. All reagents were obtained at the highest purity. The precursor Boc-dimethoxytrityl-nosyl-lyxothymidine was purchased from ABX Advanced Biomedical Compounds (Germany). The synthesis process was based on previously published methods [51] and developed by our team using Eckert & Ziegler “Modular Lab System” (Siemens Medical Solutions; Ann Arbor, MI, USA). Briefly, ^18^F-fluoride was obtained by irradiation of H_2_^18^O, concentrated by azeotropic distillation, and 25 mg of the Boc precursor in 1 mL of acetonitrile was added to the dried ^18^F-fluoride residue. The nucleophilic substitution reaction was carried out at 110 °C, and the reaction mixture was hydrolysed with 1N HCl, neutralised, and injected into the semipreparative high pressure liquid chromatography (HPLC) system (C18 column). Using H_2_O/EtOH (92/8) as mobile phase, FLT eluted at around 19 min. The product fraction was filtered into a sterile multidose vial through two 0.22 µm sterile filters. The final yield of the synthesis was usually 15% (uncorrected) with a radiochemical purity > 97%.

### Appendix A.3. FLT Quantification

For the assessment of FLT-PET studies, Patlak graphical analysis [52] was performed with an input function obtained from the image (image derived input function, IDIF). Two input functions were obtained using spherical volumes of interest (15 mm diameter) on the left ventricle (LV) and on the descending aorta (DA) in the early image (25–50 s after administration of FLT). These volumes were applied to the dynamic study to obtain, from the mean value functions, the input curves for the kinetic model [50].

The input functions were corrected for the presence of metabolites using the metabolite fractions according to the Schiepers formula: metabolite fraction = 0.42[1−exp(−0.29t)]. Patlak analysis was performed using the PMOD software (PMOD Technologies Ltd., Adliswil, Switzerland) in the time interval between 10 and 60 min after injection, determining the influx constant (Ki). Two constants were obtained, considering the input function from left ventricle (Ki_LV) and the curve from the descending aorta (Ki_DA). The dynamic study was used to obtain PET images equivalent to a static PET study at the end of the dynamic sequence corresponding to the time intervals 40–60 min and 50–60 min, which corresponds to 20- and 10-min studies starting 40–50 min after administration of the radiopharmaceutical. These images allowed for quantification of SUV_40_ and SUV_50_, respectively. SUV values were derived from the maximum uptake in the tumour.

### Appendix A.4. Gene Profiling and Validation

RNA samples were processed following manufacturer protocols (Affymetrix) and hybridised to Affymetrix Human Gene 1.0 ST Array. Microarray data analysis consisted of background correction and normalisation using RMA algorithm, and a filtering process was performed to eliminate low expression probe sets. Applying the criterion of an expression value > 32 in 50% of the samples, 23,755 probe sets were selected for statistical analysis. Linear Models for Microarray Data were used to identify the probe sets with significant differential expression. The normalisation procedure and statistical data analyses were performed with Bioconductor. Ensemble was used to identify genes belonging to the angiogenesis category. The complete microarray raw data are available through the Gene Expression Omnibus data repository.

To validate microarray data, cDNA was synthesised from total RNA using a high-capacity cDNA archive kit (Applied Biosystems; Foster City, CA, USA). Reverse transcriptase (RT) reactions contained 1 μg of RNA samples, 1× RT buffer, 4 μL of dNTPs 100 mM, 1× of random primers, 5 μL of MultiScribe RT 50 Units/μL, and 0.005 μL of RNase inhibitor 0.20 Units/μL. The reactions were incubated in GeneAmp PCR System 2400 of Applied Biosystems for 10 min at 25 °C and 2 h at 37 °C. Each cDNA sample was analysed in quadruplicate using the ViiA™ 7 Real-Time PCR System (Applied Biosystems, Carlsbad, USA). Quantitative real-time PCRs were performed using the TaqMan^®^ system. The assays used were Hs00170261_m1 (*THBS4*) and Hs 99999901_s1 (*18S*). 18S was chosen as the endogenous control for expression studies in cancer tissues because it showed the lowest variability across samples in the microarray data and had been identified as one of the most appropriate housekeeping genes for this analysis. Quantification of expression was performed relative to the endogenous control using the comparative (Δ) CT method.

## References

[1] Holmes K., Roberts O.L., Thomas A.M., Cross M.J. (2007). Vascular endothelial growth factor receptor-2: Structure, function, intracellular signalling and therapeutic inhibition. Cell. Signal..

[2] Simons M. (2012). An inside view: VEGF receptor trafficking and signaling. Physiology.

[3] Edelman M.J., Hodgson L., Wang X., Christenson R., Jewell S., Vokes E., Kratzke R. (2011). Serum vascular endothelial growth factor and COX-2/5-LOX inhibition in advanced non-small cell lung cancer: Cancer and Leukemia Group B 150304. J. Thorac. Oncol..

[4] Tsai H.L., Yeh Y.S., Chang Y.T., Yang I.P., Lin C.H., Kuo C.H., Juo S.H., Wang J.Y. (2013). Co-existence of cyclin D1 and vascular endothelial growth factor protein expression is a poor prognostic factor for UICC stage I-III colorectal cancer patients after curative resection. J. Surg. Oncol..

[5] Wang J., Guo Y., Wang B., Bi J., Li K., Liang X., Chu H., Jiang H. (2012). Lymphatic microvessel density and vascular endothelial growth factor-C and -D as prognostic factors in breast cancer: A systematic review and meta-analysis of the literature. Mol. Biol. Rep..

[6] Kristensen T.B., Knutsson M.L., Wehland M., Laursen B.E., Grimm D., Warnke E., Magnusson N.E. (2014). Anti-vascular endothelial growth factor therapy in breast cancer. Int. J. Mol. Sci..

[7] European Medical Agency Avastin®: Summary of Product Characteristics. http://www.ema.europa.eu/docs/en_GB/document_library/EPAR_-_Product_Information/human/000582/WC500029271.pdf.

[8] US Food and Drug Administration Avastin®: Prescribing Information. http://www.accessdata.fda.gov/drugsatfda_docs/label/2013/125085s263lbl.pdf.

[9] von Minckwitz G., Puglisi F., Cortes J., Vrdoljak E., Marschner N., Zielinski C., Villanueva C., Romieu G., Lang I., Ciruelos E. (2014). Bevacizumab plus chemotherapy versus chemotherapy alone as second-line treatment for patients with HER2-negative locally recurrent or metastatic breast cancer after first-line treatment with bevacizumab plus chemotherapy (TANIA): An open-label, randomised phase 3 trial. Lancet Oncol..

[10] Brufsky A.M., Hurvitz S., Perez E., Swamy R., Valero V., O’Neill V., Rugo H.S. (2011). RIBBON-2: A randomized, double-blind, placebo-controlled, phase III trial evaluating the efficacy and safety of bevacizumab in combination with chemotherapy for second-line treatment of human epidermal growth factor receptor 2-negative metastatic breast cancer. J. Clin. Oncol..

[11] Robert N.J., Dieras V., Glaspy J., Brufsky A.M., Bondarenko I., Lipatov O.N., Perez E.A., Yardley D.A., Chan S.Y., Zhou X. (2011). RIBBON-1: Randomized, double-blind, placebo-controlled, phase III trial of chemotherapy with or without bevacizumab for first-line treatment of human epidermal growth factor receptor 2-negative, locally recurrent or metastatic breast cancer. J. Clin. Oncol..

[12] Miller K., Wang M., Gralow J., Dickler M., Cobleigh M., Perez E.A., Shenkier T., Cella D., Davidson N.E. (2007). Paclitaxel plus bevacizumab versus paclitaxel alone for metastatic breast cancer. N. Engl. J. Med..

[13] Miller K.D., Chap L.I., Holmes F.A., Cobleigh M.A., Marcom P.K., Fehrenbacher L., Dickler M., Overmoyer B.A., Reimann J.D., Sing A.P. (2005). Randomized phase III trial of capecitabine compared with bevacizumab plus capecitabine in patients with previously treated metastatic breast cancer. J. Clin. Oncol..

[14] Sanghera B., Wong W.L., Sonoda L.I., Beynon G., Makris A., Woolf D., Ardeshna K. (2014). FLT PET-CT in evaluation of treatment response. Indian J. Nucl. Med..

[15] Eschmann S.M., Paulsen F., Bedeshem C., Machulla H.J., Hehr T., Bamberg M., Bares R. (2007). Hypoxia-imaging with (18)F-Misonidazole and PET: Changes of kinetics during radiotherapy of head-and-neck cancer. Radiother. Oncol..

[16] Eschmann S.M., Paulsen F., Reimold M., Dittmann H., Welz S., Reischl G., Machulla H.J., Bares R. (2005). Prognostic impact of hypoxia imaging with 18F-misonidazole PET in non-small cell lung cancer and head and neck cancer before radiotherapy. J. Nucl. Med..

[17] Hylton N. (2006). Dynamic contrast-enhanced magnetic resonance imaging as an imaging biomarker. J. Clin. Oncol..

[18] Law W.P., Miles K.A. (2013). Incorporating prognostic imaging biomarkers into clinical practice. Cancer Imaging.

[19] Carmeliet P., Pallaud C., Deurloo R.J., Bubuteishvili-Pacaud L., Henschel V., Dent R., Bell R., Mackey J., Scherer S.J., Cameron D. (2012). Plasma VEGF-A and VEGFR-2 biomarker results from the BEATRICE phase III trial of bevacizumab (BEV) in triple-negative early breast cancer. Cancer Res..

[20] Miles D.W., de Haas S.L., Dirix L.Y., Romieu G., Chan A., Pivot X., Tomczak P., Provencher L., Cortes J., Delmar P.R. (2013). Biomarker results from the AVADO phase 3 trial of first-line bevacizumab plus docetaxel for HER2-negative metastatic breast cancer. Br. J. Cancer.

[21] Gianni L., Chan A., Mansutti M., Pivot X., Greil R., Provencher L., Prot S., Moore N., Scherer S.J., Pallaud C. (2012). Biomarker results from the phase III AVEREL trial of 1st-line bevacizumab, trastuzumab plus docetaxel for HER2-positive locally recurrent/metastatic breast cancer. Ann. Oncol..

[22] Jurgensmeier J.M., Schmoll H.J., Robertson J.D., Brooks L., Taboada M., Morgan S.R., Wilson D., Hoff P.M. (2013). Prognostic and predictive value of VEGF, sVEGFR-2 and CEA in mCRC studies comparing cediranib, bevacizumab and chemotherapy. Br. J. Cancer.

[23] Wedam S.B., Low J.A., Yang S.X., Chow C.K., Choyke P., Danforth D., Hewitt S.M., Berman A., Steinberg S.M., Liewehr D.J. (2006). Antiangiogenic and antitumor effects of bevacizumab in patients with inflammatory and locally advanced breast cancer. J. Clin. Oncol..

[24] Banys-Paluchowski M., Witzel I., Riethdorf S., Pantel K., Rack B., Janni W., Fasching P.A., Aktas B., Kasimir-Bauer S., Hartkopf A. (2018). The clinical relevance of serum vascular endothelial growth factor (VEGF) in correlation to circulating tumor cells and other serum biomarkers in patients with metastatic breast cancer. Breast Cancer Res. Treat..

[25] Miles D., Cameron D., Bondarenko I., Manzyuk L., Alcedo J.C., Lopez R.I., Im S.A., Canon J.L., Shparyk Y., Yardley D.A. (2017). Bevacizumab plus paclitaxel versus placebo plus paclitaxel as first-line therapy for HER2-negative metastatic breast cancer (MERiDiAN): A double-blind placebo-controlled randomised phase III trial with prospective biomarker evaluation. Eur. J. Cancer.

[26] Liang X., Li H., Coussy F., Callens C., Lerebours F. (2019). An update on biomarkers of potential benefit with bevacizumab for breast cancer treatment: Do we make progress?. Chin J Cancer Res..

[27] Hussain S.A., Ganesan R., Reynolds G., Gross L., Stevens A., Pastorek J., Murray P.G., Perunovic B., Anwar M.S., Billingham L. (2007). Hypoxia-regulated carbonic anhydrase IX expression is associated with poor survival in patients with invasive breast cancer. Br. J. Cancer.

[28] Milani M., Harris A.L. (2008). Targeting tumour hypoxia in breast cancer. Eur. J. Cancer.

[29] Mikhaylova M., Mori N., Wildes F.B., Walczak P., Gimi B., Bhujwalla Z.M. (2008). Hypoxia increases breast cancer cell-induced lymphatic endothelial cell migration. Neoplasia.

[30] Vaupel P., Briest S., Hockel M. (2002). Hypoxia in breast cancer: Pathogenesis, characterization and biological/therapeutic implications. Wien Med. Wochenschr..

[31] Chaudary N., Hill R.P. (2006). Hypoxia and metastasis in breast cancer. Breast Dis..

[32] Rundqvist H., Johnson R.S. (2013). Tumour oxygenation: Implications for breast cancer prognosis. J. Intern. Med..

[33] Gerhardt H. (2008). VEGF and endothelial guidance in angiogenic sprouting. Organogenesis.

[34] Scherpereel A., Gentina T., Grigoriu B., Senechal S., Janin A., Tsicopoulos A., Plenat F., Bechard D., Tonnel A.B., Lassalle P. (2003). Overexpression of endocan induces tumor formation. Cancer Res..

[35] Chen L.Y., Liu X., Wang S.L., Qin C.Y. (2010). Over-expression of the Endocan gene in endothelial cells from hepatocellular carcinoma is associated with angiogenesis and tumour invasion. J. Int. Med. Res..

[36] Roudnicky F., Poyet C., Wild P., Krampitz S., Negrini F., Huggenberger R., Rogler A., Stohr R., Hartmann A., Provenzano M. (2013). Endocan is upregulated on tumor vessels in invasive bladder cancer where it mediates VEGF-A-induced angiogenesis. Cancer Res..

[37] Tian S., Roepman P., Van’t Veer L.J., Bernards R., de Snoo F., Glas A.M. (2010). Biological functions of the genes in the mammaprint breast cancer profile reflect the hallmarks of cancer. Biomark. Insights.

[38] Strasser G.A., Kaminker J.S., Tessier-Lavigne M. (2010). Microarray analysis of retinal endothelial tip cells identifies CXCR4 as a mediator of tip cell morphology and branching. Blood.

[39] Almog N., Ma L., Raychowdhury R., Schwager C., Erber R., Short S., Hlatky L., Vajkoczy P., Huber P.E., Folkman J. (2009). Transcriptional switch of dormant tumors to fast-growing angiogenic phenotype. Cancer Res..

[40] Almog N., Briggs C., Beheshti A., Ma L., Wilkie K.P., Rietman E., Hlatky L. (2013). Transcriptional changes induced by the tumor dormancy-associated microRNA-190. Transcription.

[41] Huang H., Bhat A., Woodnutt G., Lappe R. (2010). Targeting the ANGPT-TIE2 pathway in malignancy. Nat. Rev. Cancer.

[42] Singletary S.E., Allred C., Ashley P., Bassett L.W., Berry D., Bland K.I., Borgen P.I., Clark G., Edge S.B., Hayes D.F. (2002). Revision of the American Joint Committee on Cancer staging system for breast cancer. J. Clin. Oncol..

[43] National Comprehensive Cancer Network NCCN Clinical Practice Guidelines in Oncology (NCCN Guidelines®) Breast Cancer, Version 2.2016. http://www.nccn.org/professionals/physician_gls/pdf/breast.pdf.

[44] Cancer Therapy Evaluation Program Common Terminaology Criteria for Adverse Events, Version 3.0. http://ctep.cancer.gob/protocoldevelopment/electronic_applications/docs/ctcaev3.pdf.

[45] Ogston K.N., Miller I.D., Payne S., Hutcheon A.W., Sarkar T.K., Smith I., Schofield A., Heys S.D. (2003). A new histological grading system to assess response of breast cancers to primary chemotherapy: Prognostic significance and survival. Breast.

[46] Fizazi K., Morat L., Chauveinc L., Prapotnich D., De Crevoisier R., Escudier B., Cathelineau X., Rozet F., Vallancien G., Sabatier L. (2007). High detection rate of circulating tumor cells in blood of patients with prostate cancer using telomerase activity. Ann. Oncol..

[47] Tofts P.S., Brix G., Buckley D.L., Evelhoch J.L., Henderson E., Knopp M.V., Larsson H.B., Lee T.Y., Mayr N.A., Parker G.J. (1999). Estimating kinetic parameters from dynamic contrast-enhanced T(1)-weighted MRI of a diffusable tracer: Standardized quantities and symbols. J. Magn. Reson. Imaging.

[48] Cheng C., Pounds S. (2007). False discovery rate paradigms for statistical analyses of microarray gene expression data. Bioinformation.

[49] Carey V.J., Gentry J., Whalen E., Gentleman R. (2005). Network structures and algorithms in Bioconductor. Bioinformatics.

[50] Marti-Climent J.M., Dominguez-Prado I., Garcia-Velloso M.J., Boni V., Penuelas I., Toledo I., Richter J.A. (2014). [^18^F]fluorothymidine-positron emission tomography in patients with locally advanced breast cancer under bevacizumab treatment: Usefulness of different quantitative methods of tumor proliferation. Rev. Esp. Med. Nucl. Imagen Mol..

[51] Yun M., Oh S.J., Ha H.J., Ryu J.S., Moon D.H. (2003). High radiochemical yield synthesis of 3′-deoxy-3′-[18F] fluorothymidine using (5′-O-dimethoxytrityl-2′-deoxy-3′-O-nosyl-beta-D-threo pentofuranosyl)thymine and its 3-N-BOC-protected analogue as a labeling precursor. Nucl. Med. Biol..

[52] Patlak C.S., Blasberg R.G., Fenstermacher J.D. (1983). Graphical evaluation of blood-to-brain transfer constants from multiple-time uptake data. J. Cereb. Blood Flow. Metab..

