# Supplementary material for: Early Imaging and Molecular Changes with Neoadjuvant Bevacizumab in Stage II/III Breast Cancer
Source: Cancers (Basel). 2021 Jul 14;13(14):3511. doi: 10.3390/cancers13143511 (PMC8307791; doi:10.3390/cancers13143511)
Supplement: Supplementary file 1 [file cancers-13-03511-s001.zip › cancers-1161547-supplementary.pdf]

SUPPLEMENTARY MATERIAL

Supplementary Figure

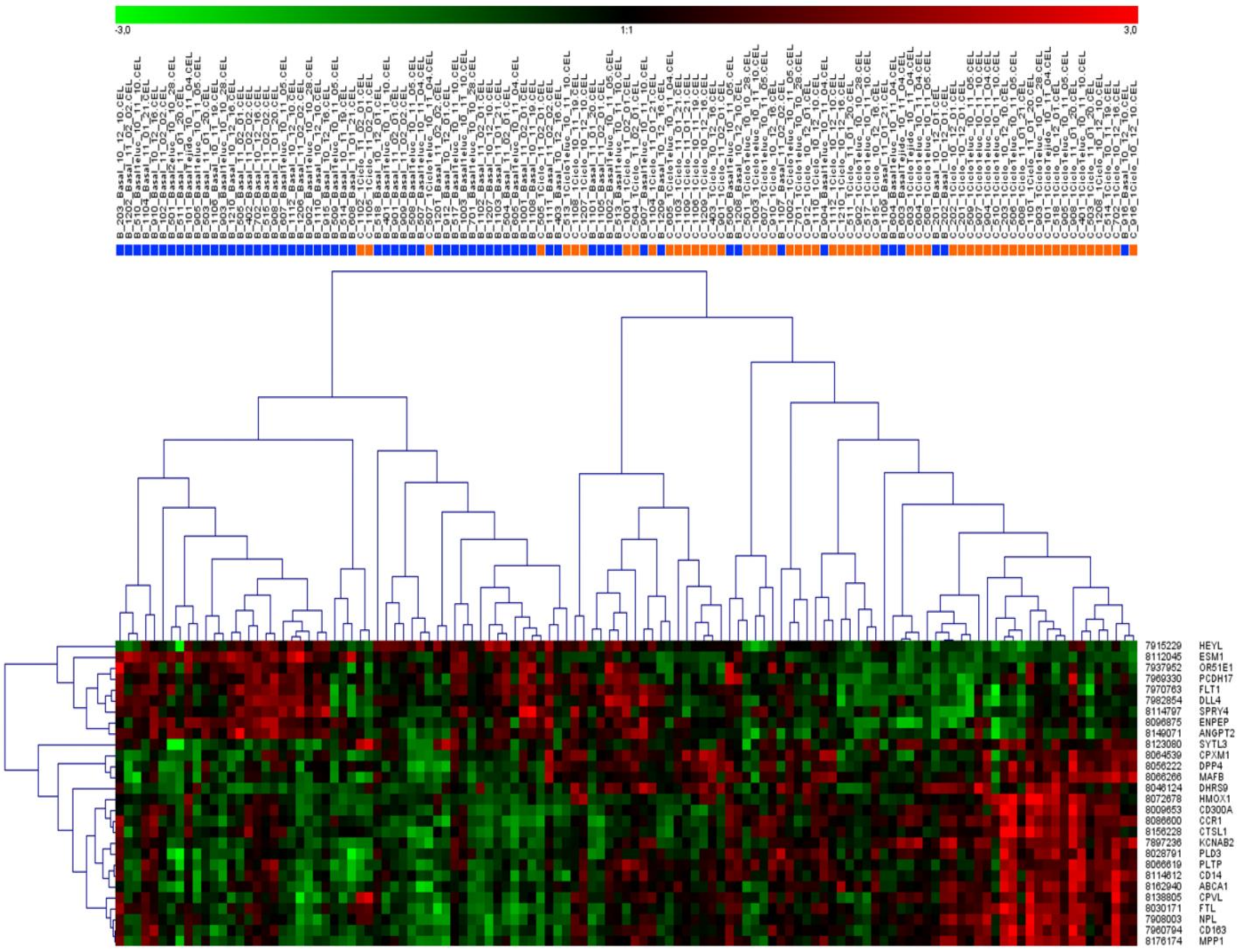

Figure S1: Hierarchical clustering of 119 samples according to expression of the top 28 differentially expressed probes (pre- and post-bevacizumab; baseline and after C1, respectively). Columns represent samples; rows represent individual probes; heat map depicts high (red) and low (green) relative levels of expression.

Supplementary Table

Table S1: Differential change in gene expression from baseline after bevacizumab treatment (after C1) using Linear Models for Microarray data.

Gene description [gene symbol]	Molecular function	Log-fold change in gene expression
Endothelial cell-specific molecule 1 [*ESM1*]	Extracellular region	–0.72
FMS-related tyrosine kinase 1 (vascular endothelial growth factor/vascular permeability factor receptor) [*FLT1*]	Angiogenesis, blood vessel development, response to hypoxia	–0.43
Angiopoietin 2 [*ANGPT2*]	Angiogenesis, blood vessel development, extracellular region, regulation of angiogenesis	–0.36
Sprouty homolog 4 [*SPRY4*]	Cell projection, plasma membrane	–0.34
Glutamyl aminopeptidase (aminopeptidase A) [*ENPEP*]	Aminopeptidase activity, angiogenesis, blood vessel development, cell motility	–0.34
Delta-like 4 [*DLL4*]	Angiogenesis, blood vessel development, cell motility, endothelial cell migration, NOTCH pathway signalling	–0.33
Olfactory receptor, family 51, subfamily E, member 1 [*OR51E1*]	Intrinsic to membrane	–0.27
Protocadherin 17 [*PCDH17*]	Intrinsic to membrane	–0.24

Hairy/enhancer-of-split related with YRPW motif-like [*HEYL*]	NOTCH pathway signalling	–0.20
Synaptotagmin-like 3 [*SYTL3*]	Not specified	+0.22
Phospholipase D family, member 3 [*PLD3*]	Intrinsic to membrane, phospholipase D activity	+0.24
Ferritin, light polypeptide [*FTL*]	Intracellular ferritin complex, iron ion homeostasis	+0.24
CD300a molecule [*CD300A*]	Intrinsic to membrane	+0.25
Potassium voltage-gated channel, shaker-related subfamily, beta member 2 [*KCNAB2*]	Cell projection, intrinsic to membrane	+0.29
Carboxypeptidase, vitellogenic-like [*CPVL*]	Carboxypeptidase activity (serine-type), peptidase activity (L-amino peptides)	+0.33
V-maf musculoaponeurotic fibrosarcoma oncogene homolog B [*MAFB*]	Erythrocyte homeostasis	+0.33
Membrane protein, palmitoylated 1, 55 kDa [*MMP1*]	Cell fraction, intrinsic to membrane	+0.38
Cathepsin 1 [*CTSL1*]	Aminopeptidase activity	+0.41
N-acetylneuraminate pyruvate lyase (dihydrodipicolinate synthase) [*NPL*]	Not specified	+0.42
CD14 molecule [*CD14*]	Cytokine production, inflammatory response, phagocytosis	+0.43
ATP-binding cassette, subfamily A (ABC1), member 1 [*ABCA1*]	Cdc42 protein signal transduction, cytokine production, phagocytosis, platelet dense granule organisation	+0.43
Chemokine (C-C motif) receptor 1 [*CCR1*]	Inflammatory response, peptide binding	+0.43
Carboxypeptidase X (M14 family), member 1 [*CPXM1*]	Metalloexopeptidase activity, peptidase activity	+0.43

Dehydrogenase/reductase (SDR family) member 9 [*DHRS9*]	9-cis-retinoic acid biosynthesis process	+0.49
Phospholipid transfer protein [*PLTP*]	Extracellular region	+0.53
Dipeptidyl-peptidase 4 [*DPP4*]	Aminopeptidase activity, endothelial cell migration, peptidase activity, regulation of leukocyte-mediated immunity, response to hypoxia	+0.56
CD163 molecule [*CD163*]	Inflammatory response	+0.59
Haem oxygenase (decycling) 1 [*HMOX1*]	Angiogenesis, blood vessel development, haem oxidation, inflammatory response, response to hypoxia	+0.84
